# An illustrated key to the Saudi Arabian species of the genus *Macroocula* Panfilov, 1954, with the description of a new species and the previously unknown female of *M. andreai* Pagliano (Hymenoptera, Bradynobaenidae, Apterogyninae)

**DOI:** 10.3897/zookeys.742.22854

**Published:** 2018-03-12

**Authors:** Ahmed M. Soliman, Neveen S. Gadallah, Hathal Mohammed Al Dhafer

**Affiliations:** 1 Plant Protection Department, College of Food and Agriculture Sciences, King Saud University, P.O. BOX 2460, Riyadh 11451, Saudi Arabia; 2 Zoology Department, Faculty of Science (Boys), Al-Azhar University, P.O. Box 11884, Nasr City, Cairo, Egypt; 3 Entomology Department, Faculty of Science, Cairo University, Giza, Egypt

**Keywords:** Asir, Jazan, *Macroocula*, new species, Saudi Arabia

## Abstract

The Saudi Arabian species of the genus *Macroocula* Panfilov (Bradynobaenidae, Apterogyninae) are keyed and illustrated. Eleven species were previously recorded from Arabian fauna: *M. andreai* Pagliano (♂), *M. atuberculata* Soliman & Gadallah (♂), *M. khorimensis* Soliman & Gadallah (♂), *M. magna* (Invrea) (♀), *M. mahunkai* (Argaman) (♂), *M. nitida* (Bischoff) (♂, ♀), *M. ohli* Pagliano (♂), *M. riyadha* Gadallah & Pagliano (♂), *M. savignyi* (Klug) (♂, ♀), *M. sinaica* (Invrea) (♂) and *M. zulfiensis* Soliman & Gadallah (♂). A new species, *Macroocula asirensis* Gadallah & Soliman, **sp. n.** (♂) from Saloos Al-Manzar, Wadi Yebah and Wadi Targ (Asir region) and the previously unknown female of *M. andreai* from Wadi Reem (Jazan region) are described and illustrated.

## Introduction

The genus *Macroocula* Panfilov, 1954 is considered one of the largest of the subfamily Apterogyninae (Hymenoptera, Bradynobaenidae), representing about 27 % of the total number of apterogynine species (Pagliano 2002, 2008, 2011, Gadallah et al. 2014, 2015, Soliman et al. 2016). Species are distributed in Africa and Asia, predominantly in the Afrotropical region (Pagliano 2002, 2008, 2011; Gadallah et al. 2014; Soliman et al. 2016). Only a single species, *M. savignyi* (Klug) is also reported from India (Dover 1924).

*Macroocula* species are mainly characterised by their peculiarly large eyes and their dull colour that are correlated with their nocturnal habits (Pagliano 2002, 2008). No biological studies were done concerning this genus, so the biology of the genus as well as of the whole Apterogyninae is still largely unknown.

The genus *Macroocula* is represented in the Arabian Peninsula countries by the following number of species based on Pagliano (2002, 2008, 2011), Gadallah et al. (2014) and Soliman et al. (2016): Kuwait (no species records), Oman (seven species), Saudi Arabia (eleven species), United Arab Emirates (four species), and Yemen (five species).

In Saudi Arabia, Pagliano (2002) reported five *Macroocula* species. Gadallah et al. (2014) added three species, of which one, *M. riyadha* Gadallah & Pagliano, was described as new. Recently, further three new species were described from Riyadh region, *M. atuberculata* Soliman & Gadallah, *M. khorimensis* Soliman & Gadallah and *M. zulfiensis* Soliman & Gadallah (Soliman et al. 2016).

In the present study, *M. asirensis* Gadallah & Soliman, sp. n. (Asir region) is described and illustrated, thus raising the total number of species to twelve. In addition, the previously unknown female of *M. andreai* Pagliano is described for the first time. An illustrated key to all *Macoocula* species known from Saudi Arabia, including the male genitalia, is provided.

## Materials and methods

The present study is based on specimens collected from different regions in Saudi Arabia: **Asir region** (Saloos Al-Manzar, Wadi Baqrah, Wadi Targ, Wadi Yabah); **Jazan region** (Ahad Al-Masareha, Muhaiel–Al-Darb Road, Wadi Jizan, Wadi Reem, Wadi Shahadam); **Riyadh region** (Al-Khararah, Al-Wasiel, Hawtet Bani Tameem, Ibex Reserve, Rawdhet Al-Harmalyiah, Rawdhet Al-Sabalh, Rawdhet Farshet Sheaal, Rawdhet Khorim, Wadi Ghaihab, Wadi Hanifah, Wadi Haradah) . A male specimen of *Macroocula ohli* from Wadi Muqshin, Dhofar (**Oman**) and a male specimen of *Macroocula nitida* from Wadi Garawi, southern of Helwan (**Egypt**) were also examined. The specimens were collected using light and pitfall traps or hand-picked at night, and are deposited in the King Saud University Museum of Arthropods (**KSMA**), Plant Protection Department, College of Food and Agriculture Sciences, King Saud University, Riyadh, Saudi Arabia, and Efflatoun Bey collection (**EFC**), Entomology Department, Faculty of Science, Cairo University, Giza, Egypt.

The male genitalia were removed from metasoma using dissecting pins, then placed in cold NaOH 10 % for 24 hours (except in *M. ohli*, the photo was taken while the genitalia was still attached to the specimen) and washed in distilled water prior passing in 70–100 % ethanol series for dehydration. For photography, the genitalia were fixed in glycerin jelly on microscopic slides.

Morphological terminology is based on Pagliano (2002). Body sculpture terminology follows Harris (1979). Photographic images were taken using a Canon EOS 70D camera attached to a Leica MZ 125 stereomicroscope. Individual source images were then stacked using HeliconFocus v. 6.22 (HeliconSoft Ltd) extended depth of field software. Measurements of the different parts were made with the help of an ocular micrometer. Further image processing was done using the software Adobe Photoshop CS5.1 (v. 12.1x32).

### Abbreviations

**F1, F2, F3, etc.** first, second, third, etc. antennal flagellomeres.

**IOD** interocellar distance.

**LED** longitudinal eye diameter.

**MOD** mid ocellus diameter.

**MS** malar space

**OOD** ocellocular distance.

**S1, S2, S3, etc.** first, second, third, etc. metasomal sternites.

**T1, T2, T3, etc.** first, second, third, etc. metasomal tergites.

## Taxonomy

### Genus Macroocula

Taxon classificationAnimaliaHymenopteraBradynobaenidae

Panfilov, 1954

Macroocula Panfilov, 1954: 149, type species: Apterogyna morawitzi Radoskowski, 1888, by original designation.
Doryleika Argaman, 1994: 46, type species: Doryleika mahunkai Argaman, 1994, by original designation. Junior subjective synonym of Macroocula Panfilov, 1954 by Pagliano 2002: 125.

#### Diagnosis.

Eyes bulged, hemispherical, with its diameter about 4× distance between inner eye margin and antennal tubercle in male, and equal to distance between inner eye margin and antennal tubercle in female; distance between eye and occipital carina at most equal to eye diameter in female and by less than its own diameter in male; ocelli larger than or at least as large as flagellar diameter; hind trochanter without protuberance as that of mid trochanter in most *Macroocula* species, except for few Egyptian and Arabian species; posterior margin of metasomal tergites without fringe of setae, except for the Arabian species *M. riyadha* (Pagliano 2002, 2008, Gadallah et al. 2014, 2015, Soliman et al. 2016).

#### Key to the species of the genus *Macroocula* in SA (except *M. mahunkai*)

**Table:** 

1	Wingless, female (unknown for *asirensis* sp. n., *atuberculata*, *khorimensis*, *ohli*, *riyadha*, *sinaica*, *zulfiensis*, *mahunkai*)	**2**
–	Winged, male (unknown for *magna*)	**5**
2	Body uniformly yellow (Fig. 1A)	***M* . *andreai* Pagliano**
–	Head, mesosoma and first metasomal segment dark yellow to ferruginous-red, remaining metasomal segments black to brown or yellowish-brown (Figs 2A, 3A)	**3**
3	Head and mesosoma ferruginous-red (Fig. 2A); F1 distinctly longer than F2 (about 1.35×) (Fig. 2B); T2 wider than T3 and sparsely punctate, punctures small, rounded and 2–4 diameters apart (Fig. 2C); T6 with 13–15 interrupted longitudinal ridges, laterally with about 20 evenly spaced and nearly one-size sharp teeth (Fig. 2D); S1 abruptly swollen subapically (Fig. 2E), with median longitudinal ridge on posterior half	***M. magna* (Invrea)**
–	Head and mesosoma dark yellow to pale brown (Fig. 3A); F1 at most scarcely longer than F2 (Fig. 3B); T2 as wide as T3 and densely punctate, punctures large, ellipsoid and at most 1 diameters apart (Fig. 3C); T6 with 8–10 interrupted longitudinal ridges, laterally with about 15 sharp teeth, basal ones larger and widely spaced than apical teeth (Fig. 3D); S1 gently convex throughout, without median longitudinal carina (Fig. 3E)	**4**
4	Punctures on T3 circular, considerably smaller than those on T2, superficial and widely scattered (2–4 diameters apart) (Fig. 3C)	***M* . *savignyi* (Klug)**
–	Punctures on T3 rather ellipsoid, similar to those on T2 or slightly smaller, deeper and subcontiguous (1–2 diameters apart)	***M* . *nitida* (Bischoff)**
5	Body uniformly yellow, T2 and T3 sometimes slightly darker (Fig. 4A, B)	**6**
–	Head, mesosoma and first metasomal segment dark yellow to ferruginous-red or red, remaining metasomal segments black to brown or yellowish-brown (Figs 6A, 8A, 9A, 10A)	**7**
6	Body dark yellow (Fig. 4A); T1 subconical, as long as its maximal width or scarcely longer (Fig. 4A); hind trochanter without ventral protuberance (Fig. 4C); aedeagus distinctly shorter than parameres; parameres with an acute flange laterally at the level of cuspis (Fig. 12A)	***M. andreai* Pagliano**
–	Body pale yellow (Fig. 4B); T1 subcylindrical, about 1.35× as long as its maximal width (Fig. 4B); hind trochanter with well-developed ventral protuberance (Fig. 4D); aedeagus scarcely shorter than parameres; parameres obtusely angled at the level of cuspis (Fig. 12B) ***M. ohli* Pagliano**	***M. ohli* Pagliano**
7	Head bicolored, black and red; T3–6 posteriorly with fringe of silvery setae	***M* . *riyadha* Gadallah & Pagliano**
–	Head uniformly yellow to ferruginous-red; metasomal tergites posteriorly at most sparsely setose, without fringe of silvery setae	**8**
8	Mid and hind trochanters with well-developed protuberances (Fig. 5E)	**9**
–	Mid trochanter only with a well-developed protuberance, sometimes extremely weak or absent (as in *atuberculata*), hind trochanter evenly arched, without protuberance (Figs 6C, 8D, 10E)	**10**
9	Body including face of the head punctate-reticulate (Fig. 5A, C, F); T1 subconical, slightly longer than wide (about 1.25×) (Fig. 5F); T2 subrounded, as long as wide or slightly wider (Fig. 5F); aedeagus slightly shorter than parameres (Fig. 12C)	***M* . *khorimensis* Soliman & Gadallah**
–	Face smooth on upper half except laterally, punctulate on lower half (Fig. 5B), other parts of the body more or less sparsely punctate (Fig. 5D, G); T1 subcylindrical, distinctly longer than wide (about 1.4×) (Fig. 5G); T2 subconical, slightly longer than wide (Fig. 5G); aedeagus as long as parameres (Fig. 12D)	***M* . *zulfiensis* Soliman & Gadallah**
10	Head and mesosoma dark ferruginous-red (Fig. 6A); T1 and hind femur (except basally) dark brown (Fig. 6A); mid trochanter without protuberance in most specimens, or at most indicated as a very weak process at the apex of trochanter (Fig. 6C); parameres sharply tapering apically (Fig. 12E)	***M. atuberculata* Soliman & Gadallah**
–	Head, mesosoma, T1 and hind femur yellow to bright ferruginous-red (Figs 7C, 8A, 9A, 10A); mid trochanter always with a well-developed protuberance (Figs 8D, 9B, 10E); parameres more or less rounded apically (Fig. 12F–H)	**11**
11	Malar space relatively long, 1.0× MOD and as long as F1 width (Fig. 7B); mesoscutum strongly convex, strongly sloping forwardly (Fig. 7A); S2 evenly convex; aedeagus distinctly shorter than parameres (about 0.5× as long as parameres), slightly longer than cuspis	***M. nitida* (Bischoff)**
–	Malar space shorter, 0.3–0.5× MOD and 0.5–0.75× F1 width (Figs 8B, 9A); mesoscutum slightly convex, gently sloping forwardly (Fig. 9A); S2 basally more or less depressed (Fig. 8F); aedeagus as long as parameres or slightly shorter (Fig. 12F–H)	**12**
12	Malar space 0.5 MOD (Fig. 8B, C); T1 subconical, as long as wide or scarcely longer (1.16×) (Fig. 8E); T2 strongly convex laterally, wider than long (about 0.9× as long as wide), with rounded and deep punctures (Fig. 8E); aedeagus slightly shorter than parameres, which is thick on apical half (Fig. 12F)	***M* . *sinaica* (Invrea)**
–	Malar space 0.3 MOD (Figs 9A, 10B); T1 subcylindrical, distinctly longer than wide (about 1.4×) (Figs 9A, 10A); T2 less convex laterally, longer than wide (about 1.1× as long as wide) (Figs 9C, 11B), with ellipsoid shallow punctures with longitudinal ridges in between (Figs 9C, 11B); aedeagus as long as parameres, which are narrow (finger-like) on apical half (Fig. 12G, H)	**13**
13	Head, mesosoma and first metasomal segment bright ferruginous-red (Fig. 9A); protuberance on mid trochanter lobe-like, blunt apically (Fig. 9B); T2 abruptly sloping posteriorly, with wide punctures and short ridges in between (Fig. 9C); S1 foveate (Fig. 9D); S6 laterally with dense and stout red bristles, distinctly different from the adjacent pale setae (Fig. 9E); parameres laterally with distinct flange at the level of cuspis (Fig. 12G)	***M* . *savignyi* (Klug)**
–	Head, mesosoma and first metasomal segment yellow (Fig. 10A); protuberance on mid trochanter triangular, pointed apically (Fig. 10E); T2 gently sloping posteriorly, with punctures longitudinally stretched (narrow) and long ridges in between (Fig. 11B); S1 transversely ridged (Fig. 11C); S6 laterally with sparse, fine, pale bristles, hardly differentiated from the adjacent pale setae (Fig. 10F); parameres without lateral flange, evenly rounded (Fig. 12H)	***M. asirensis* sp. n.**

**Figure 1.**
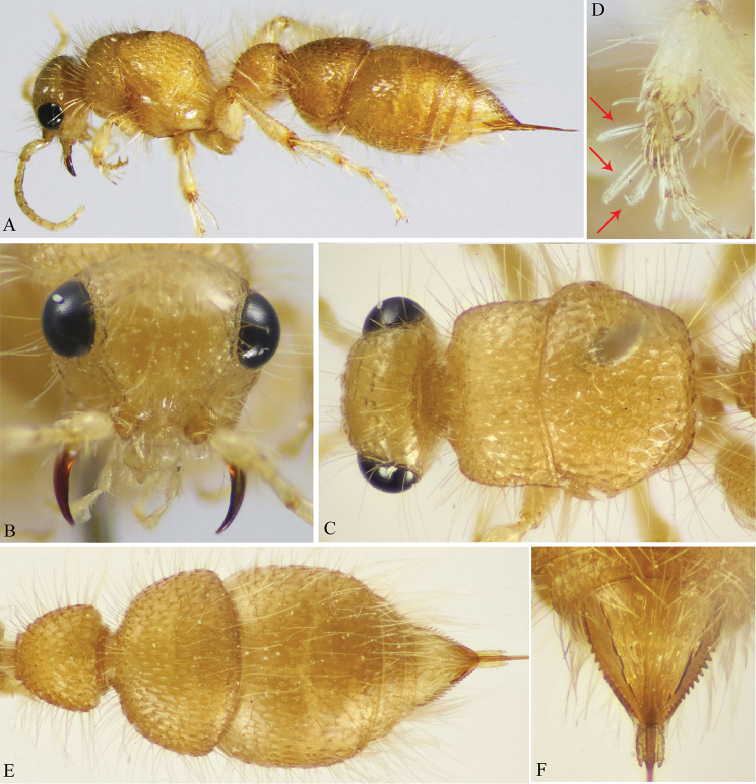
**A–F** *M. andreai* Pagliano (♀). **A** Habitus, dorso-lateral view **B** Head, frontal view **C** Head & mesosoma, dorsal view **D** Fore tibia & tarsus (tarsal spatulate spines indicated) **E** Metasoma, dorsal view **F** Pygidium (T6).

**Figure 2.**
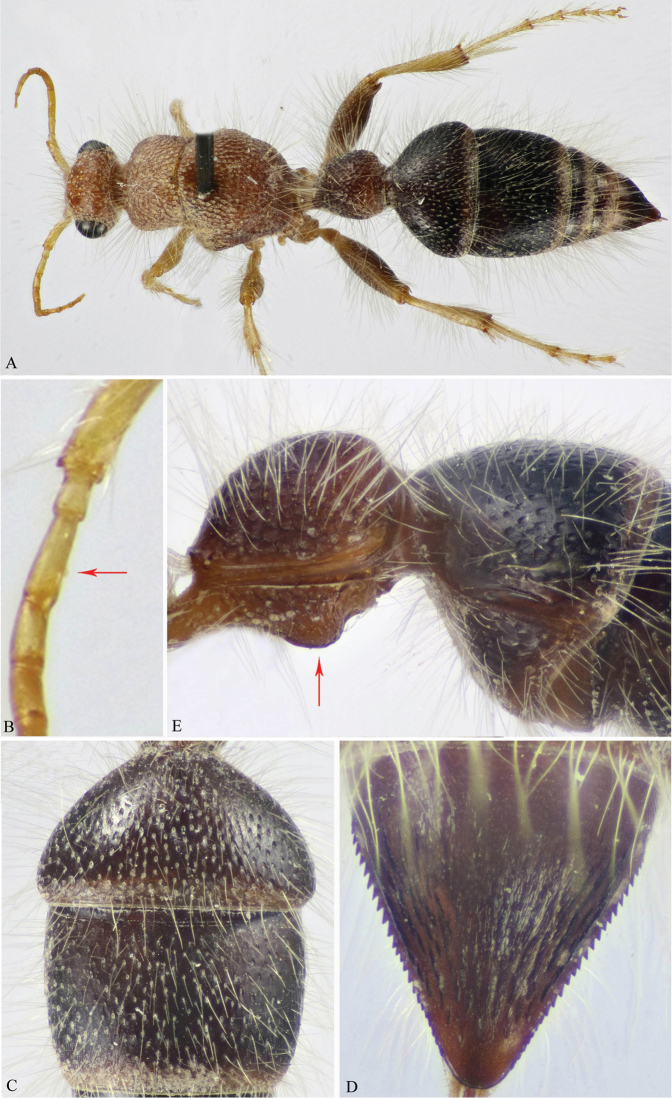
**A–E** *M. magna* (Invrea) (♀). **A** Habitus, dorsal view **B** Scape (part), pedicel & F1–3 (F1 indicated) **C** T2 & T3 **D** Pygidium (T6) **E** First and second metasomal segments, lateral view (swelling on S1 indicated).

**Figure 3.**
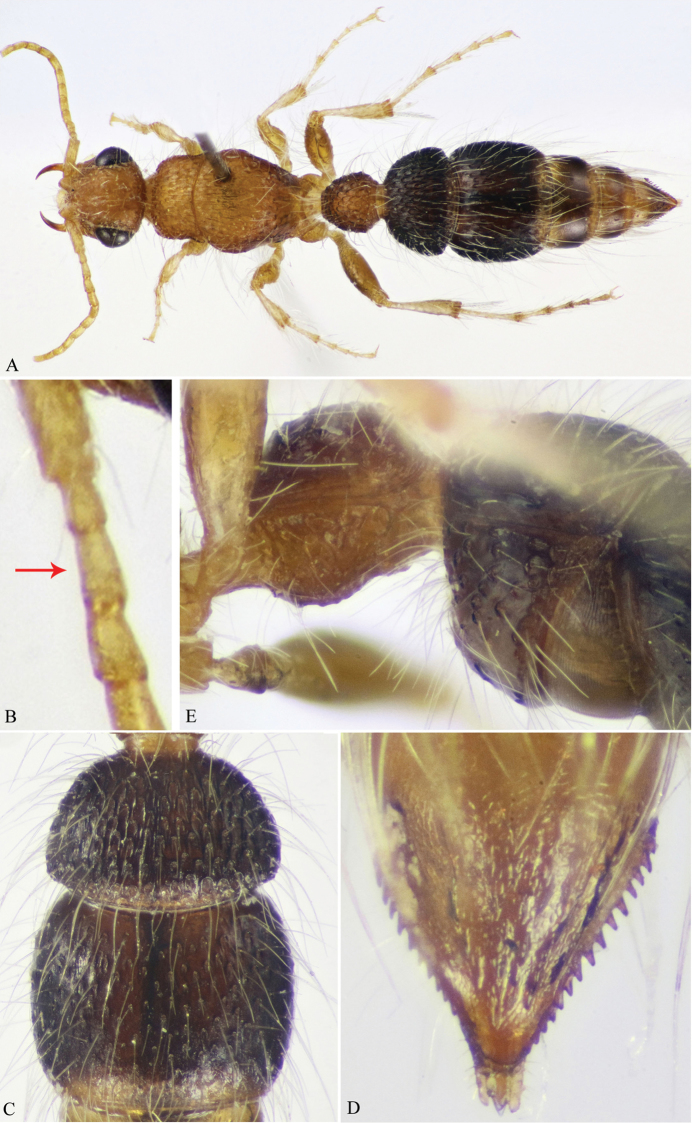
**A–E** *M. savignyi* (Klug) (♀). **A** Habitus, dorsal view **B** Scape (part), pedicel & F1–2 (F1 indicated) **C** T2 & T3 **D** Pygidium (T6) **E** First and second metasomal segments, latero-ventral view.

**Figure 4.**
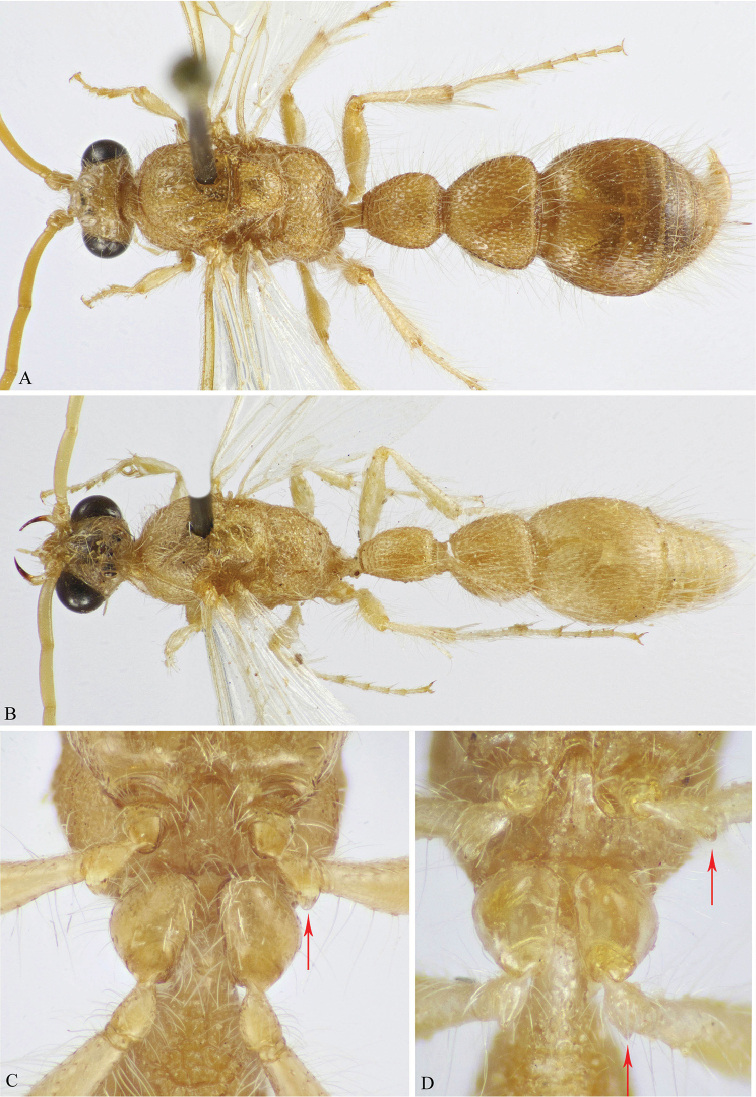
**A, C** *M. andreai* Pagliano (♂) **B, D** *M. ohli* Pagliano (♂) **A, B** Habitus, dorsal view **C, D** Mid and hind coxae and trochanters (protuberance on trochanters indicated).

**Figure 5.**
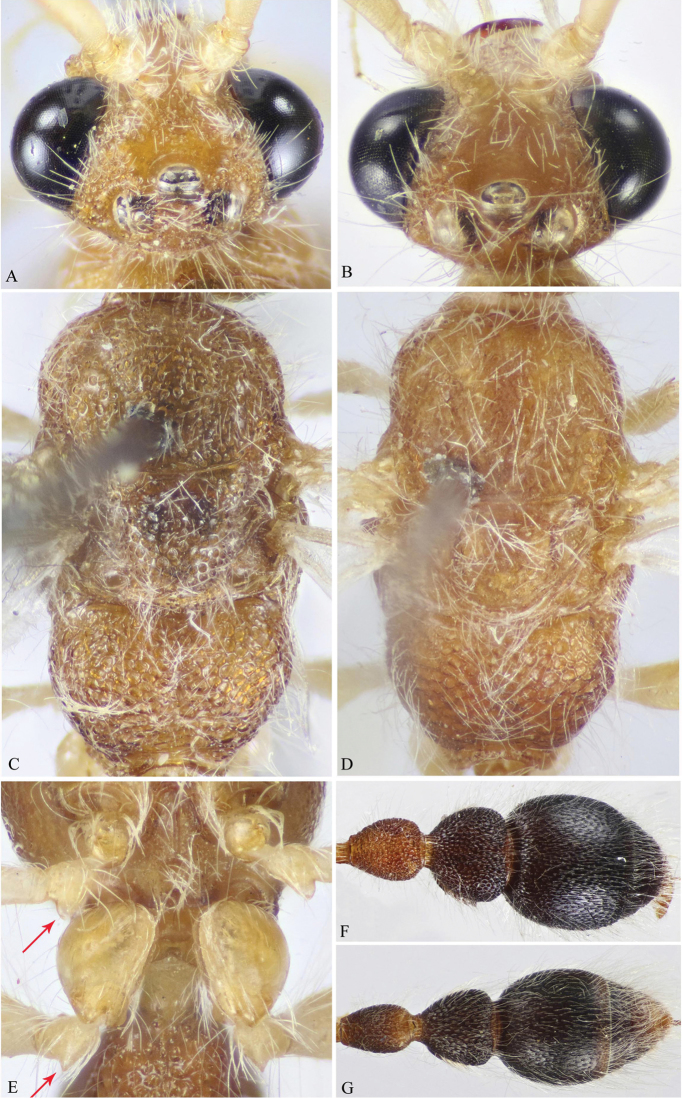
**A, C, E, F** *M. khorimensis* Soliman & Gadallah (♂) **B, D, G** *Macroocula zulfiensis* Soliman & Gadallah (♂) **A, B** Head, dorsal view **C, D** Mesosoma, dorsal view **E** Mid and hind coxae and trochanters (protuberance on trochanters indicated) **F & G** Mesosoma, dorsal view.

**Figure 6.**
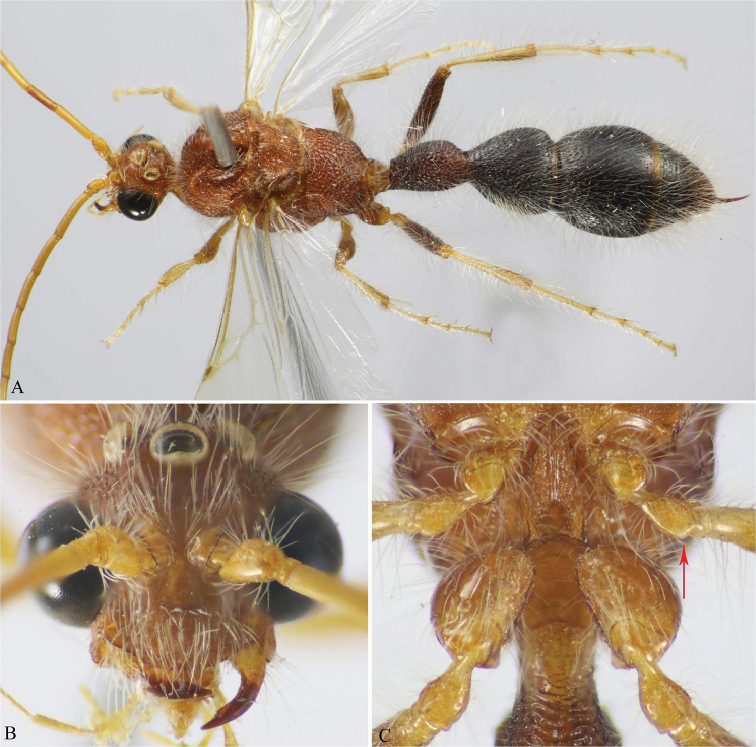
**A–C** *M. atuberculata* Soliman & Gadallah (♂). **A** Habitus, dorsal view **B** Head, frontal view **C** Mid and hind coxae and trochanters (weak protuberance on mid trochanter indicated).

**Figure 7.**
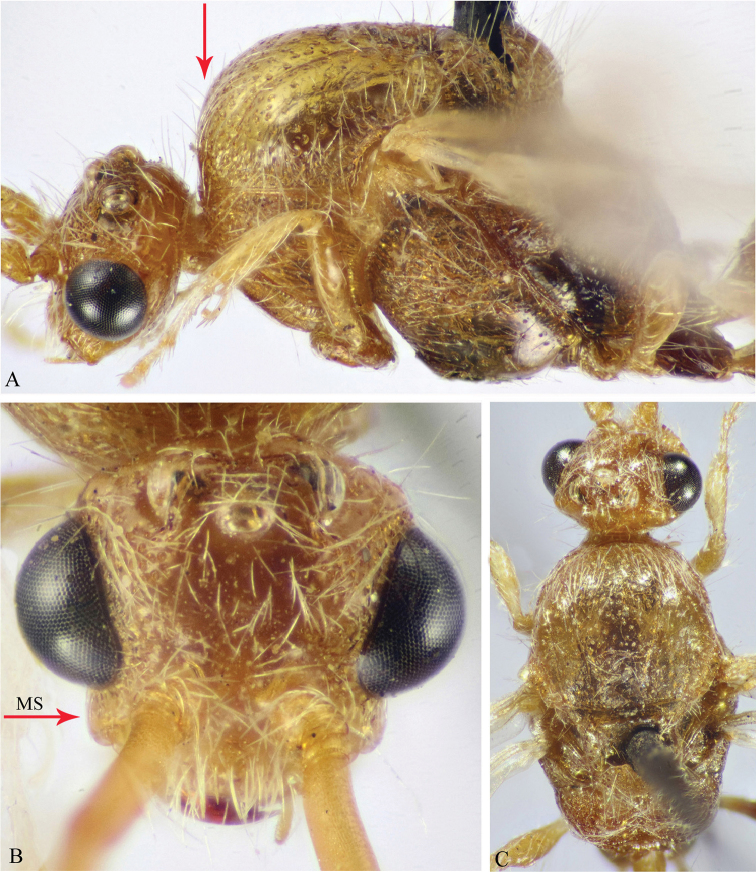
**A–C** *M. nitida* (Bischoff) (♂). **A** Head and mesosoma, dorso-lateral view (strong sloping of mesoscutum indicated) **B** Head, frontal view (malar space indicated) **C** Head and mesosoma, dorsal view.

**Figure 8.**
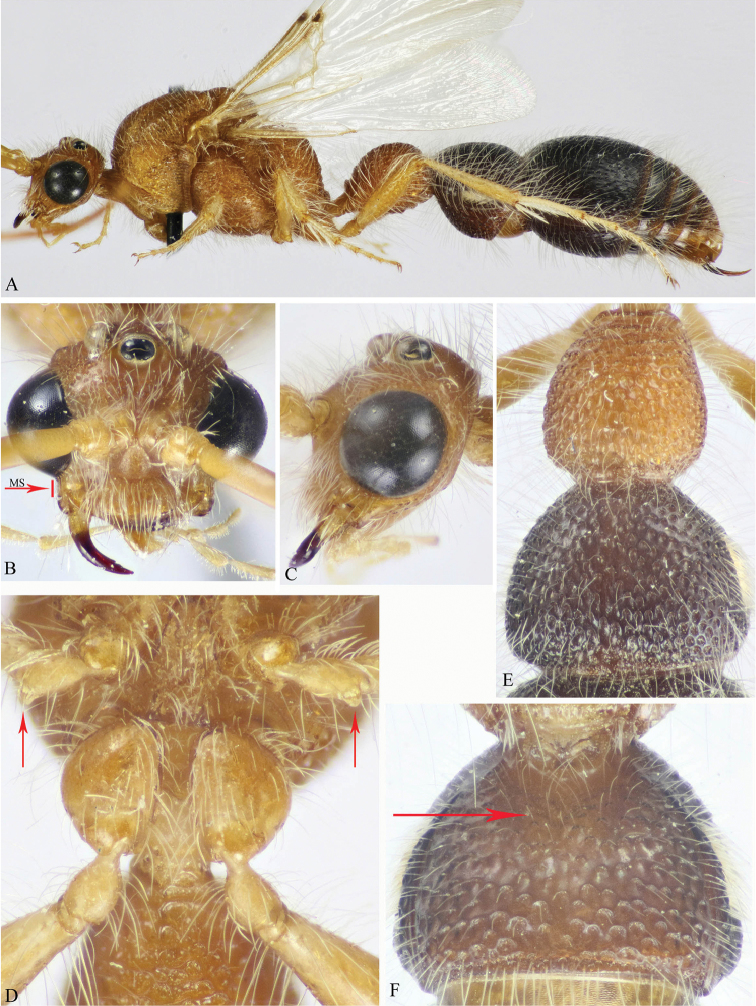
**A–F** *M. sinaica* (Invrea) (♂). **A** Habitus, lateral view **B, C** Head, frontal & lateral views respectively **D** Mid and hind coxae and trochanters (protuberance on mid trochanter indicated) **E** T1 & T2 **F** S2 (basal depression indicated).

**Figure 9.**
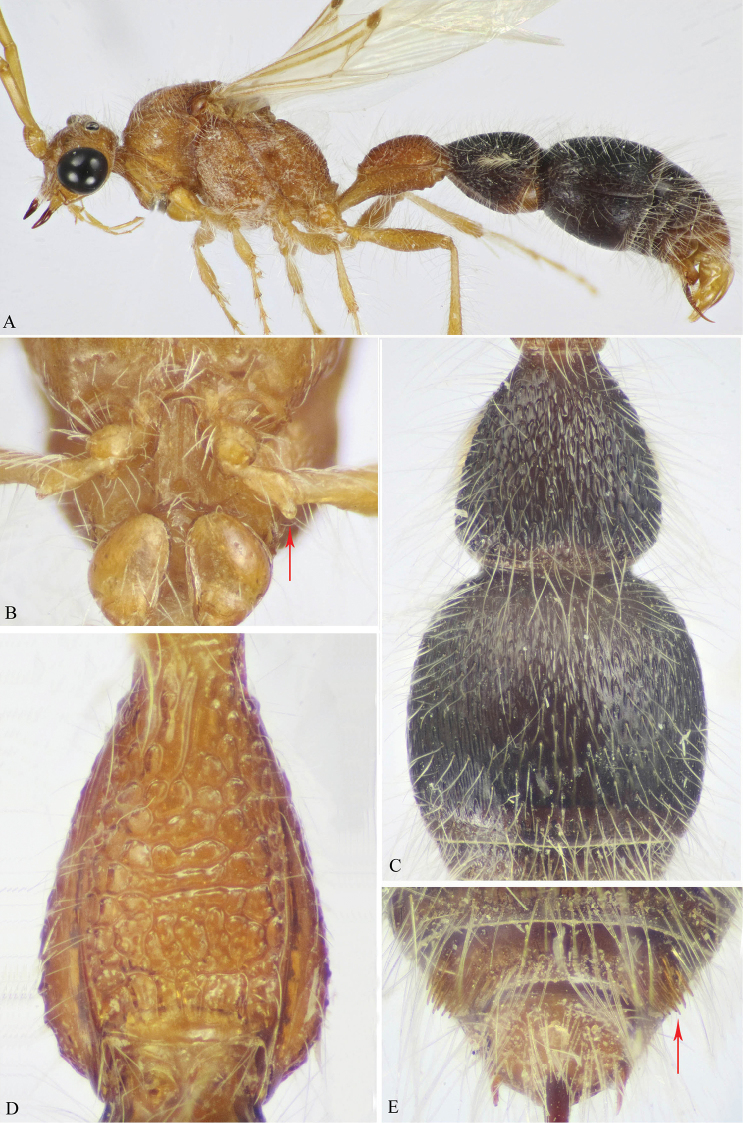
**A–E** *M. savignyi* (Klug) (♂). **A** Habitus, lateral view **B** Mid and hind coxae and mid trochanter (protuberance on mid trochanter indicated) **C** T1 & T2 **D** S1 **E** S6–7 (lateral bristles on S6 indicated).

##### List of newly described species

### Macroocula asirensis

Taxon classificationAnimaliaHymenopteraBradynobaenidae

Gadallah & Soliman sp. n.

http://zoobank.org/1362CD8F-FB1D-452C-9533-DD9CC628C743

**Figure 10.**
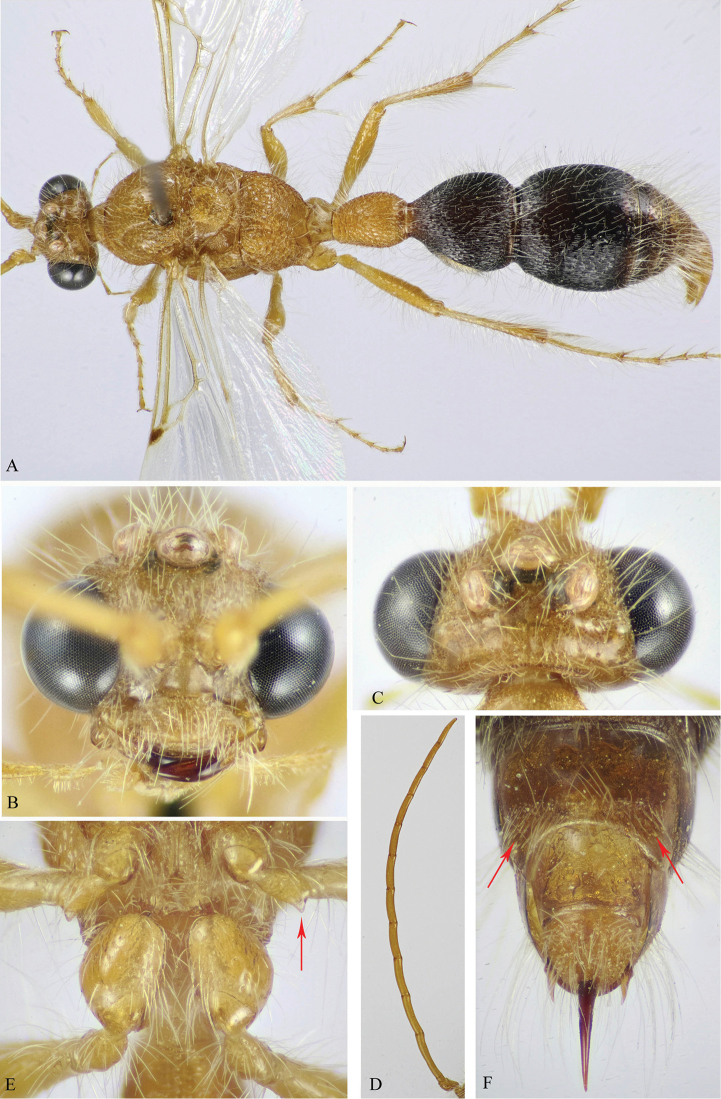
**A–F** *M. asirensis* sp. n. (holotype ♂). **A** Habitus, dorsal view **B & C** Head, frontal & dorsal views respectively **D** Antenna **E** Mid and hind coxae and trochanters (protuberance on mid trochanter indicated) **F** S6–7 (lateral bristles on S6 indicated).

**Figure 11.**
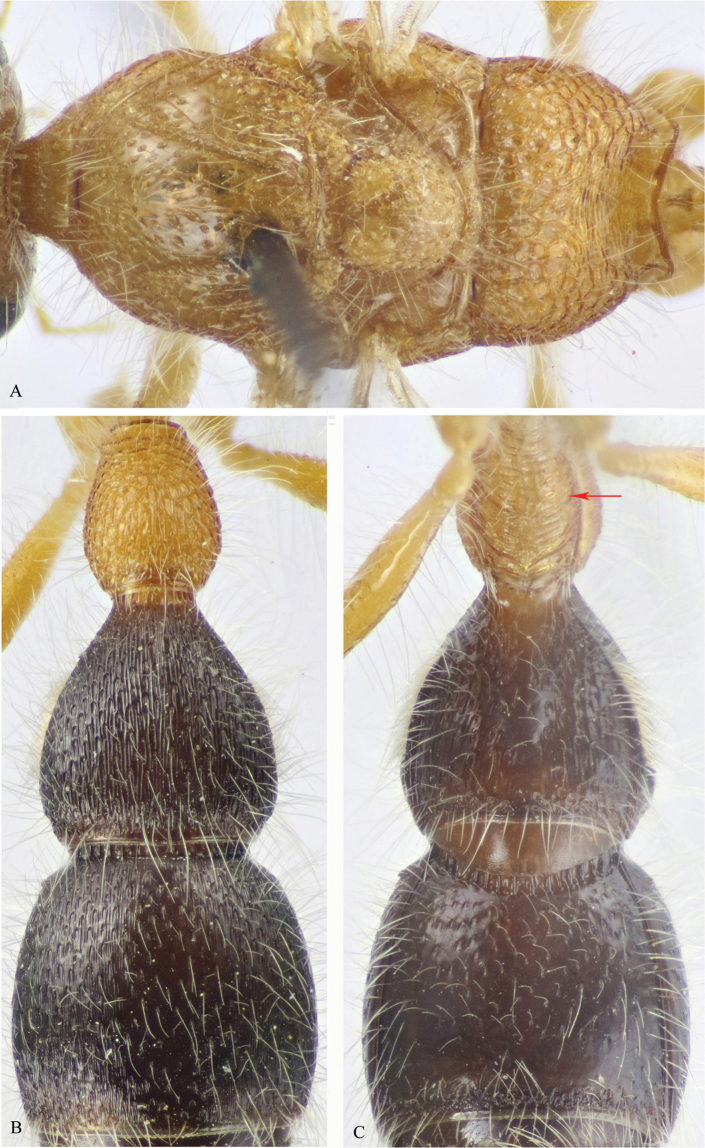
**A–C** *M. asirensis* sp. n. (holotype ♂). **A** Mesosoma, dorsal view **B** T1–3 **C** S1–3.

**Figure 12.**
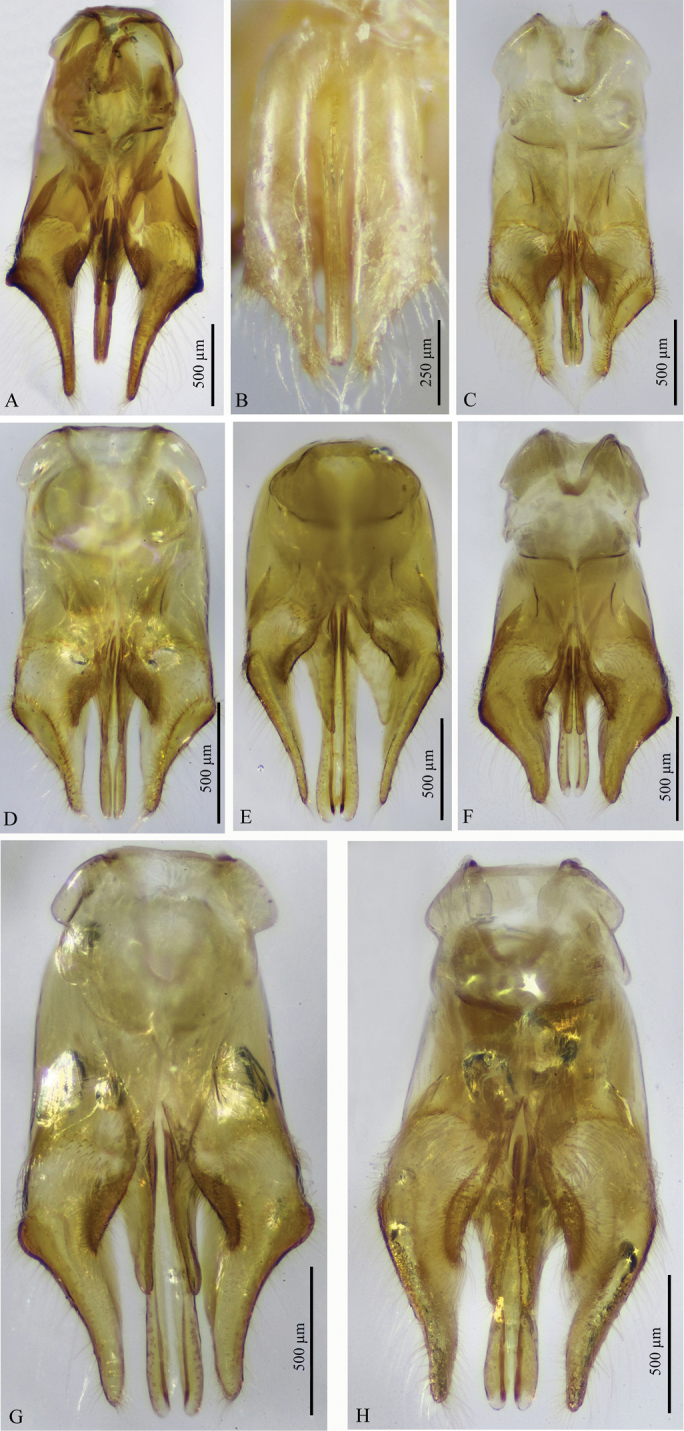
**A–H** Male genitalia of *Macroocula* species (ventral view except for *M. ohli*, dorsal view). **A** *M. andreai* Pagliano **B** *M. ohli* Pagliano **C** *M. khorimensis* Soliman & Gadallah **D** *M. zulfiensis* Soliman & Gadallah **E** *M. atuberculata* Soliman & Gadallah **F** *M. sinaica* (Invrea) **G** *M. savignyi* (Klug) **H** *M. asirensis* sp. n. (paratype).

Figs 10A–F , 11A–C , 12H

#### Material examined.

Holotype ♂: Saudi Arabia, Saloos Al-Manzar, Wadi Baqrah (**Asir**) [18°47'30"N, 42°01'05"E, Alt. 422 m], light trap, 4.XI.2013, leg. Al Dhafer et al. [**KSMA**]. Paratypes 1♂: Saudi Arabia, Almagardah, Wadi Yebah (**Asir**) [19°16'16"N, 41°48'38"E, Alt. 402 m], light trap, 12.III.2012, leg. Abdeldayem M. & Eltorkey A. [**KSMA**]; 1♂: Saudi Arabia, Saloos Al-Manzar, Wadi Baqrah (**Asir**) [18°47'24"N, 41°58'18"E, Alt. 331 m], light trap, 13.III.2012, Abdeldayem M. & Eltorkey A. [**EFC**]; 1♂: Saudi Arabia, Wadi Targ (**Asir**) [19°37'22"N, 42°18'01"E, Alt. 1317 m], light trap, 14.III.2012, leg. Al Dhafer H. & Fadl H. [**KSMA**]; 1♂: Saudi Arabia, Almagardah, Wadi Yebah (**Asir**) [19°14'53"N, 41°47'15"E, Alt. 402 m], light trap, 11.X.2013, leg. Khan S. [**KSMA**].

#### Diagnosis.

Head, mesosoma and first metasomal segment yellow; mid trochanter with triangular protuberance, pointed apically; T2 gently sloping posteriorly, with longitudinally stretched, narrow punctures, the interspaces with long ridges; S1 transversely ridged; lateral bristles on S6 sparse, fine and pale in colour, hardly differentiated from adjacent pale setae; parameres without lateral flange, evenly rounded at the level of cuspis.

#### Description.

MALE (holotype). Body length 16.5 mm; fore wing length 12 mm.

*Colour* (Figs 10A–F, 11C). Head including antenna and basal half of mandible, mesosoma and first and last visible (the 7^th^) metasomal segments yellow; remaining segments of metasoma brown to black, 6^th^ and 7^th^ segments yellowish; apical half of mandible and median terminal hook reddish-brown (chestnut); palpi and legs yellow; fore tibial spur yellow, mid and hind spurs waxy white; wings hyaline, fore wing with veins pale yellow and stigma reddish. Body including legs and scape of antenna densely clothed with long, erect to suberect pale setae; S2–S6 apically with fringe of long erect setae restricted to median third of the sternites; S6 laterally with sparse, fine, pale bristles.

*Head* (Fig. 10A–D). Dorsally as wide as maximal width of mesosoma at mesopleuron; face and vertex finely sparsely punctate, except along inner ocular orbit punctures are denser; vertex slightly swollen postero-laterally; ocelli distinctly large; OOD about 0.4× IOD; malar space distinctly short, about 0.3× MOD; clypeus convex, with free margin straight; gena nearly smooth, with widely scattered punctures along the outer ocular orbit; mandible slender, pointed apically and edentate (simple); scape of antenna slightly shorter than wide; F1 5× as long as wide, 1.2× as long as F2; F3 as long as F2.

*Mesosoma* (Figs 10E, 11A). Pronotum foveate-reticulate dorsolaterally, with lateral face horizontally ridged; mesoscutum and scutellum sparsely punctate, punctures 1-2 diameters apart, denser at borders; mesoscutum with longitudinal median smooth stripe and complete notauli that are widely divergent anteriorly; tegula smooth; propodeum foveate-reticulate on dorsal and lateral faces, posterior face transversely ridged. Mesopleuron foveate-reticulate dorsally, sparsely punctate ventrally; metapleuron smooth below spiracle; metasternum with three longitudinal carinae “median carina is the strongest”, bidentate in front of hind coxae, space between denticles U-shaped. Fore wing with brachial cell as long as wide, scarcely shorter than anterior vein of cubital cell; hind wings with 8 hamuli. Mid trochanter ventrally with well developed, triangular (pointed apically) protuberance, hind trochanter evenly arched, without a protuberance.

*Metasoma* (Figs 10F, 11B, C). T1 subcylindrical, distinctly longer than its maximal width (1.35×), irregularly rugose; T2 pear-shaped, slightly longer than its maximal width (1.12×), gently sloping posteriorly, with punctures strongly stretched (narrow), the interspaces with long ridges; T3 as T2, punctures 1–2 diameters apart on disc; T4–T7 punctulate; S1 transversely ridged; S2 medially depressed along basal two-thirds, laterally largely ellipsoid punctate; S3 sparsely punctate, with double row of punctures along posterior margin; S4–S6 finely punctate-subreticulate; S6 laterally with sparse, fine, pale bristles, hardly distinguished from adjacent pale setae.

*Genitalia* (Fig. 12H). Generally densely setose, gently widened medially, about 1.9× as long as its maximal width; parameres finger-like on apical third, rounded at apex, as long as aedeagus, densely setose along its inner and outer sides as well as in the area surrounding volsella; cuspis of volsella in ventral view lobe-like, densely setose along its whole surface; digitus distinctly longer than cuspis and about 0.6× as long as aedeagus, with scattered setae on outer face.

**Female.** Unknown.

#### Etymology.

The species epithet *asirensis* refers to Asir region where the specimens were collected.

#### Distribution.

Saudi Arabia (Asir region).

#### Remarks.

The new species, *Macroocula asirensis* sp. n., is very similar to *M. savignyi* (Klug) from which it differs mainly in the following aspects: head, mesosoma and first metasomal segment yellow (Fig. 10A) (in *savignyi* darker, ferruginous-red: Fig. 9A); protuberance on mid trochanter pointed apically (Fig. 10E) (blunt in *savignyi*: Fig. 9B); T2 gently sloping at posterior margin, with longitudinally stretched, narrow punctures, the interspaces with long ridges (Fig. 11B) (in *savignyi*, T2 abruptly sloping posteriorly, with normal punctures and short ridges in between: Fig. 9C); S1 transversely ridged (Fig. 11C) (foveate in *savignyi*: Fig. 9D); lateral bristles on S6 sparse, fine and pale in colour, hardly differentiated from adjacent pale setae (Fig. 10F) (in *savignyi* dense, stout and dark in colour “red”, distinctly differentiated from adjacent pale setae: Fig. 9E); parameres without lateral flange (Fig. 12H) (in *savignyi* parameres laterally with rounded flange at the level of cuspis: Fig. 12G); genitalia densely setose on parameres and cuspis (in *savignyi* setae noticeably less dense); digitus of volsella with scattered setae on outer face but still denser than in *savignyi*.

### Macroocula andreai

Taxon classificationAnimaliaHymenopteraBradynobaenidae

Pagliano, 2002

Female: Fig. 1A–F ; Male: Figs 4A, C , 12A

Macroocula andreai Pagliano, 2002: 144.

#### Material examined.

1♀: Saudi Arabia, Wadi Reem (**Jazan**) [17°52'39"N, 42°16'33"E, Alt. 196 m], hand picking at night, 18.XI.2015, leg. Ahmed M. Soliman [**KSMA**]; 55♂: Saudi Arabia, Wadi Reem (**Jazan**) [17°52'39"N, 42°16'33"E, Alt. 196 m], light trap, 18.XI.2015, leg. Ahmed M. Soliman [**KSMA**]; 2♂: Saudi Arabia, Wadi Reem (**Jazan**) [17°52'39"N, 42°16'33"E, Alt. 196 m], light trap, 18.XI.2015, leg. Ahmed M. Soliman [**EFC**]; 7♂: Saudi Arabia, Wadi Reem (**Jazan**) [17°52'36"N, 42°16'50"E, Alt. 196 m], light trap, 23.X.2016, leg. Ahmed M. Soliman [**KSMA**]; 19♂: Saudi Arabia, Muhaiel–Al-Darb Road (**Jazan**) [17°55'27"N, 42°15'20"E, Alt. 200 m], light trap, 3.IV.2017, leg. Ahmed M. Soliman [**KSMA**].

#### Description

**(Female)**. Body length 8.8 mm.

*Colour* (Fig. 1A–F). Entirely clear yellow including basal third of mandible, antennae, and legs; apical two thirds of mandible dark reddish-brown; eyes black, labrum and palpi very pale yellow. Body including legs densely clothed with long erect pale setae; fore tibial spur pale yellow, mid and hind spurs waxy white; ovipositor dark yellow, ovipositor sheath darker.

*Head* (Fig. 1A–C). Dorsally 0.65× as wide as maximal width of mesosoma, vertex and lower face superficially punctate, punctures apart by a distance equal to puncture diameter; frons smooth, impunctate; clypeus feebly convex, with straight apical margin; gena nearly smooth, with very few (hardly seen) punctures near to outer ocular orbit; malar space about 0.7× as long as LED; mandible stout, sickle-shaped, simple, sharply pointed apically; antennal tubercles subquadrate, distance between them about as long as tubercle width; minimum interocular distance 1.7× LED; antenna with scape distinctly long, 3.1× as long as broad, F1 1.5× as long as broad, F1 as long as F2 and F3, last flagellomere 2.4× as long as broad, truncate apically; maxillary palp relatively long, densely setose, 6-segmented, labial palp 4-segmented.

*Mesosoma* (Fig. 1A, C, D). Pronotal dorsal surface rectangular (excluding anterior collar), 2× as wide as its length, densely superficially foveate, humeral angle rounded and posterior margin broadly concave, lateral face of pronotum with few, very weak horizontal ridges; rest of mesosomal dorsum with dense, somewhat deeper fovea, that increase in size posteriorly. Propodeal posterior face declivous, smooth; mesepimeron densely coarsely punctate, mesepisternum finely punctate; metapleuron smooth. Fore leg with basitarsus with three unequal spatulate spines along its external edge; hind femur somewhat swollen above, densely punctate at outer margin; hind basitarsus distinctly long, about 2× as long following tarsomere, claws with a small basal blunt tooth; inner hind tibial spur slightly longer than outer spur.

*Metasoma* (Fig. 1A, E, F). First segment short, T1 subquadrate, progressively widened posteriorly, slightly wider than long at maximum width (1.1× as wide as long), superficially foveate; T2 semispherical, distinctly wider than long (1.3×), with considerable basal constriction or short petiole, punctulate, punctures elongate, one diameter distant from each other, integument smooth in between, much denser laterally; T3 is the largest, very superficially, sparsely punctate, denser laterally, T4 and T5 superficially punctate; T6 (epipygium) with distinctly longitudinal interrupted ridges (about 9 in number), lateral ridges are the strongest, lateral teeth are strong centrally, becoming minute at distal end. S1–2 sparsely punctate; S3–4 smooth, with single row of small punctures along posterior margin; S5 smooth except laterally with fine sparse punctures.

**Male (Figs 4A, C, 12A).** Body length 9.0–14 mm. Similar to female except for the following: Body somewhat darker, at least on metasoma, terminal hook and lateral spines dark ferruginous; mandible stouter than in female, with two minute subapical dents; antennal scape distinctly short, flagellomeres distinctly long and cylinder, F1 7.0× as long as broad, and slightly longer than F2 (1.16×); apical margin of clypeus depressed and rounded; mesopleuron foveate throughout, metapleuron horizontally rugate; fore wing with brachial cell rectangular, and equal in size to the anterior vein of cubital cell; hind wing with 7–10 hamuli; hind trochanter atuberculate, mid trochanter with large, lobe-like tubercle; metasomal T1 conical shaped, about as long as wide, both T1 and T2 with dense deep foveolation, somewhat oval in T2; T3 with longitudinal punctures that are opened and confluent with each other posteriorly giving the appearance of longitudinal ridges.

#### Distribution.

Saudi Arabia (Pagliano 2002).

#### Remarks.

The female of *M. andreai* was collected from Wadi Reem (Jazan) during its walking near to the light trap, while several tens of males were attracted to the light. It greatly resembles that of *M. yemenita* (Invrea), but differs in the following: body yellow (pale yellow in *yemenita*); frons of head smooth, impunctate, superficially punctate on lower face (frons coarsely punctate in *yemenita*); antennal F1 and F2 are equal-sized (F1 slightly shorter than F2 in *yemenita*); basitarsus with the usual three spatulate spines along outer edge (only two in *yemenita*).

It is also similar to *M. sajia* Pagliano, but differs in the following: pronotum transverse, 2.0× as wide as long (1.5× in *sajia*); lateral face of pronotum with few, extremely weak horizontal ridges (with vertical striae that are punctate in between in *sajia*); mesepimeron coarsely punctate, mesepisternum finely punctate, metapleuron smooth (meso- and metapleura with horizontal striae in *sajia*); T6 with distinct longitudinal interrupted ridges (T6 almost smooth, with some weak ridges towards distal margin, hardly distinct except under high magnification in *sajia*).

## Supplementary Material

XML Treatment for Macroocula

XML Treatment for Macroocula asirensis

XML Treatment for Macroocula andreai
